# Longitudinal *in vivo* evaluation of bone regeneration by combined measurement of multi-pinhole SPECT and micro-CT for tissue engineering

**DOI:** 10.1038/srep10238

**Published:** 2015-05-19

**Authors:** Philipp S. Lienemann, Stéphanie Metzger, Anna-Sofia Kiveliö, Alain Blanc, Panagiota Papageorgiou, Alberto Astolfo, Bernd R. Pinzer, Paolo Cinelli, Franz E. Weber, Roger Schibli, Martin Béhé, Martin Ehrbar

**Affiliations:** 1 Laboratory for Cell and Tissue Engineering, Department of Obstetrics, University Hospital Zurich, Schmelzbergstr. 12, 8091 Zurich, Switzerland; 2 Center for Radiopharmaceutical Sciences ETH-PSI-USZ, Paul Scherrer Institute, OIPA/103, 5232 Villigen, Switzerland; 3 Swiss Light Source, Paul Scherrer Institute, 5232 Villigen, Switzerland; 4 Division of Trauma Surgery, Center for Clinical Research, University Hospital Zurich, Sternwartstrasse 14, 8091 Zurich, Switzerland; 5 Department of Cranio-Maxillofacial Surgery, Oral Biotechnology and Bioengineering, University Hospital Zurich, Frauenklinikstrasse 24, 8091 Zurich, Switzerland; 6 Department of Chemistry and Applied Biosciences, ETH Zurich, 8092 Zurich, Switzerland

## Abstract

Over the last decades, great strides were made in the development of novel implants for the treatment of bone defects. The increasing versatility and complexity of these implant designs request for concurrent advances in means to assess *in vivo* the course of induced bone formation in preclinical models. Since its discovery, micro-computed tomography (micro-CT) has excelled as powerful high-resolution technique for non-invasive assessment of newly formed bone tissue. However, micro-CT fails to provide spatiotemporal information on biological processes ongoing during bone regeneration. Conversely, due to the versatile applicability and cost-effectiveness, single photon emission computed tomography (SPECT) would be an ideal technique for assessing such biological processes with high sensitivity and for nuclear imaging comparably high resolution (<1 mm). Herein, we employ modular designed poly(ethylene glycol)-based hydrogels that release bone morphogenetic protein to guide the healing of critical sized calvarial bone defects. By combined *in vivo* longitudinal multi-pinhole SPECT and micro-CT evaluations we determine the spatiotemporal course of bone formation and remodeling within this synthetic hydrogel implant. End point evaluations by high resolution micro-CT and histological evaluation confirm the value of this approach to follow and optimize bone-inducing biomaterials.

Regeneration and healing of large bone defects still is a clinical problem, which in significant numbers of patients desires the implantation of autologous bone tissue or the application of bone inducing materials^1^. As these treatment options require the harvest of bone from a second site or the use of large doses of osteoinductive factors, such as bone morphogenetic protein (BMP), there is a great demand for novel biomaterial-based treatment regimens^2^. In order to optimize the performance of such healing inducing biomaterials it is important to carefully follow the stability of the biomaterial, its infiltration by osteogenic cells, the formation of novel bone and the remodeling of the premature bone to become fully functional and load-bearing^3^.

All these criteria can easily be monitored on harvested tissue specimen. Histological evaluations using color stains visualizing cellular and matrix components, mineral deposition by calcein stains or bromodesoxyuridine (BrdU)-based stains to assess proliferation are well established^4^. However, since bone formation is a process which takes a couple of weeks and bone remodeling is only fully completed after many weeks, the appropriate longitudinal sampling of tissue requires a large number of animals and is a time- and labor-intensive process^5^. Therefore, techniques that allow for longitudinal non-invasive assessment of bone formation are desirable. Among such techniques, micro-computed tomography (micro-CT) has been broadly established for *in vivo* detection of bone tissue^6^,^7^. Moreover, reduction of animal numbers by longitudinal non-invasive imaging not only comes with ethical and economic benefits, but as well significantly improves the quality of obtained data due to the ability to follow the regenerative process in the same animal^8^. While for such *in vivo* evaluations micro-CT analysis with good resolution has become available^9^, for the monitoring of biomaterial stability and cell invasion, techniques with adequate resolution are still being developed^10^.

Single photon emission computed tomography (SPECT) and positron emission tomography (PET) are clinical routine techniques for the evaluation of bone metastasis and its metabolic activity using ^99m^Tc-bisphosphonate complexes (for SPECT) or ^18^F-fluoride (for PET)^11^,^12^. Since probes which accumulate in areas of active bone deposition and remodeling are available for SPECT and PET, both techniques hold great promise for the use in monitoring bone tissue regeneration in patients or in preclinical animal models even in settings where micro-CT evaluations are difficult to conduct due to lack of contrast^13^. PET imaging using ^18^F-fluoride offers advantages such as high sensitivity and accurate quantification (2 mm resolution)^14^. SPECT evaluations using Technetium-99m-labeled methylene diphosphonate (^99m^Tc-MDP) in combination with a recently developed multiplexed multi-pinhole technology provide artefact-free images with higher resolution (down to 0.5 mm) and high sensitivity in imaging smaller objects like mice^15^.

Here, we set out to establish a SPECT and CT based evaluation procedure for the longitudinal *in vivo* evaluation of bone-inducing biomimetic matrices that provides reliable and fast spatial information on bone formation and remodeling activities and therefore allows the ideal timing of sample collection. As a test matrix we employed soft (94 ± 25 Pa shear modulus)^16^, enzymatically cross-linked poly(ethylene glycol) (PEG)-based matrix metalloproteinase-1 (MMP-1) sensitive hydrogels decorated with 50 μM cell adhesion peptide (RGD)^17^,^18^. This provisional matrix was earlier shown to promote the regeneration of bone defects when supplemented with bone morphogenetic protein 2 (BMP-2)^16^. Critical size calvarial defects were generated in mice and immediately treated with preformed PEG-based hydrogels that contained or did not contain BMP-2. To generate longitudinal co-registered SPECT and micro-CT datasets, five animals per condition were injected with ^99m^Tc-MDP and scanned two hours post-injection at multiple subsequent time points post operation (weeks 1, 2, 4, 6, 9 and 12). SPECT and micro-CT data were processed using customized semi-automated scripts, which allowed both standardization of SPECT images and co-localization of metabolic and bone mineral signals. The comparison of longitudinal evaluations with histological and micro-CT evaluations at end points indicates that the longitudinal *in vivo* evaluation by SPECT and micro-CT holds great promise for the determination of bone formation and bone remodeling as is required for the efficient optimization of novel biomaterials regarding degradability, stiffness or release of growth factors.

## Results

### Biomaterial-induced bone regeneration by BMP-presenting hydrogel implants

**Figure 1:**
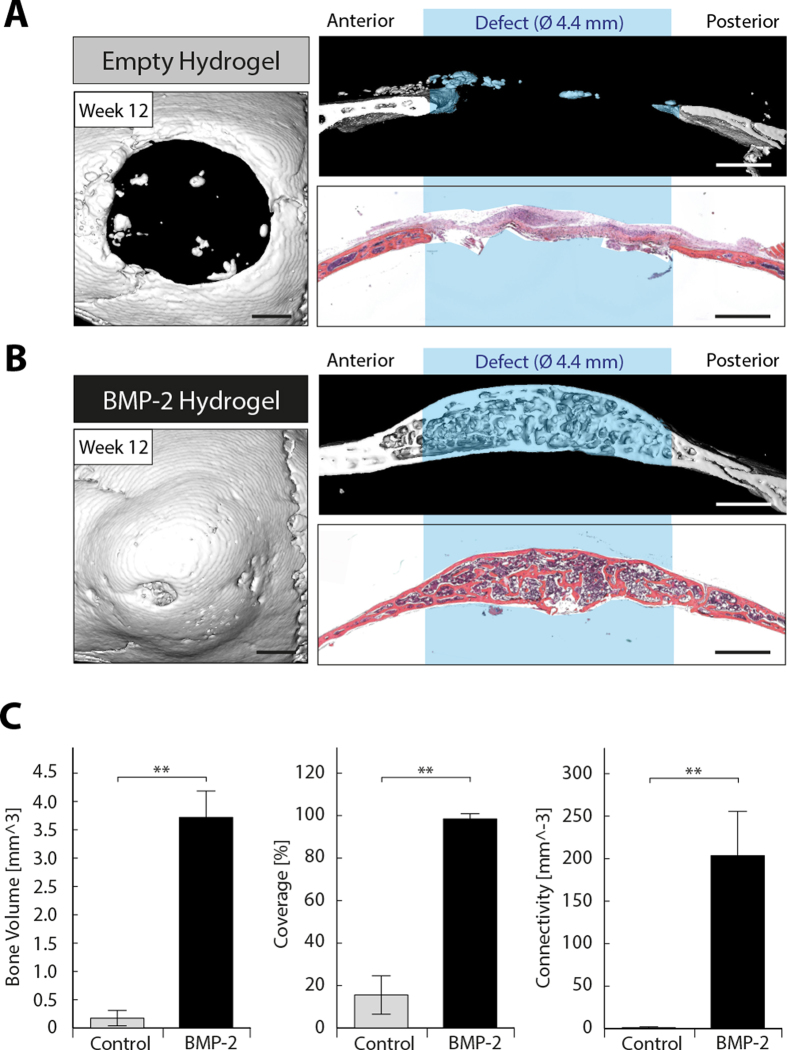
Calvarial defect regeneration by BMP-2 presenting synthetic hydrogel implants. Bone defects of approximately 4.4 mm diameter were created in the cranium of C57Bl/6 mice. Preformed hydrogel implants with a diameter of 4.4 mm and a height of 1.25 mm were positioned in the defect and bone regeneration was assessed *ex vivo* 12 weeks post-operation. Representative top views and sagittal views of 3D surface rendered micro-CT measurements as well as hematoxylin and eosin stained sections of a sagittal cross section for empty control hydrogels (**A**) and hydrogels loaded with 2 μg BMP-2 (**B**). **C**) Quantitative analysis of parameters for bone regeneration (bone volume, defect coverage, and connectivity) in defects treated with control hydrogels (gray bars) and with BMP-2-containing hydrogels (black bars). Scale bars = 1 mm. Data is depicted as mean ± SD (n = 5, ** = p < 0.01).

In order to assess biomaterial-induced bone formation and remodeling, a preclinical murine fracture model was established. Critical-sized bone defects (ca. 4.4 mm in diameter) were generated in the calvarium of C57BL/6 mice and treated with previously developed synthetic PEG-based hydrogel implants, termed ‘TG-PEG hydrogels’^17^,^18^. The stiffness and degradability of the hydrogels were tuned such that they served as permissive scaffolds for cells contributing to defect regeneration^16^. However, although the hydrogels were completely remodeled 12 weeks post-operation, no significant bone formation was observed in the defects as assessed by *ex vivo* micro-CT and histological evaluation (Fig. 1A).

Delivery of recombinant biomolecules, such as bone morphogenetic proteins (BMPs), was widely shown to serve as potent mean to foster bone regeneration^19^,^20^. Indeed, addition of 2 μg BMP-2 to the TG-PEG hydrogels led to complete healing of the bone defects 12 weeks after implantation (Fig. 1B). Bone volume increased from 0.2 ± 0.1 mm^3^ in the empty implants to 3.7 ± 0.4 mm^3^ in the BMP-2 containing implants. Furthermore, bone coverage increased from 16 ± 8% to 98 ± 2% and connectivity density from 1 ± 1 mm^-3^ to 203 ± 46 mm^-3^ (Fig. 1C).

### Longitudinal *in vivo* assessment of biomaterial-induced bone formation

**Figure 2:**
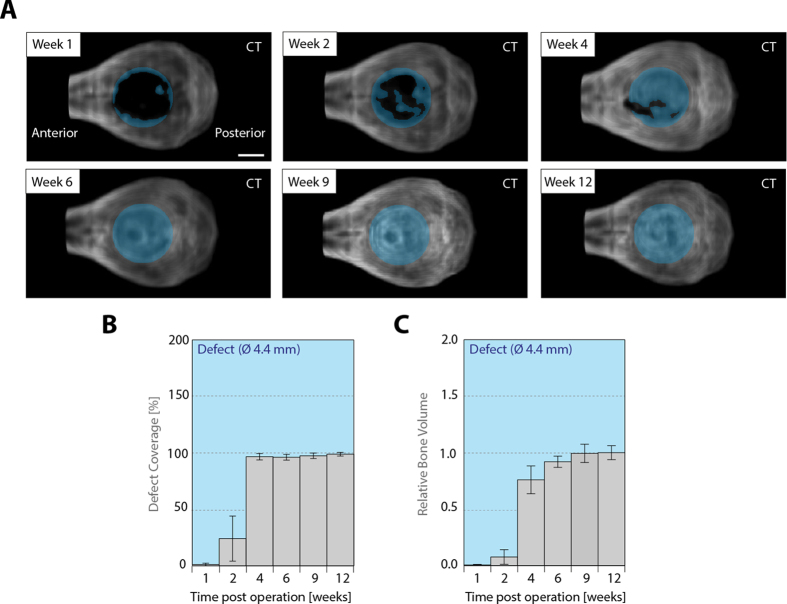
Longitudinal *in vivo* assessment of bone regeneration induced by BMP-2-presenting hydrogels. Preformed BMP-2 presenting hydrogels (Ø 4.4 mm, ht. 1.25 mm) were placed in critical-sized calvarial defects (ca. Ø 4.4 mm) in C57BL/6 mice. **A**) Top views of 3D volume rendered micro-CT *in vivo* measurements of a representative animal at indicated time points after surgery. The defect location is indicated by a blue shaded circle. **B**) Longitudinal *in vivo* assessment of the coverage of the defect and **C**) bone volume in the defect by micro-CT. Scale bar = 2 mm. Data is depicted as mean ± SEM (n = 5).

Since implant-induced bone repair is dependent on the ability of cells to populate and remodel an implant, the kinetic and spatiality of mineral deposition within the implant are important parameters to characterize and optimize novel implants. In order to monitor the progress of bone formation within the BMP-2 loaded TG-PEG hydrogels, treated mice were longitudinally and non-invasively assessed by micro-CT measurements at 6 time points, namely 1, 2, 4, 6, 9, and 12 weeks after implantation (Fig. 2A). 1 week after implantation, there was no notable mineral deposition detected within the defects. However, 2 weeks post-implantation, the defects were already substantially covered with novel bone and after 4 weeks the defects were almost fully covered with a layer of bone mineral (Fig. 2B). Over the following 8 weeks the plural stayed closed. Interestingly, similar dynamics were observed for the gain of bone volume in the defect (Fig. 2C). Bone volume steeply increased between 2 and 4 weeks of treatment and changed only marginally thereafter.

### ^99m^Tc-MDP as tracer to monitor hydroxyapatite formation

**Figure 3:**
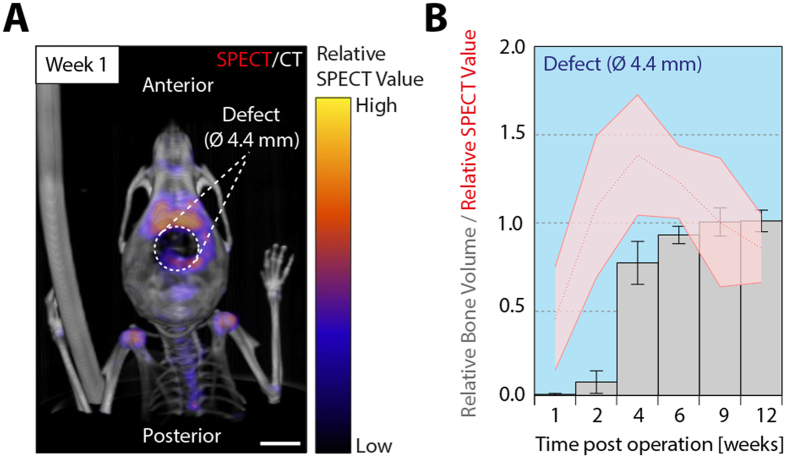
^99m^Tc-MDP accumulates at sites of bone regeneration. ^99m^Tc-MDP distribution was monitored *in vivo* 2 hours post-injection by SPECT at indicated time points after implantation of BMP-2-presenting hydrogels. Simultaneously, *in vivo* micro-CT measurements were performed for 3D anatomical orientation and to assess bone volume. **A**) Overlay of 3D volume rendered micro-CT (grayscale) and SPECT (pseudo colored) measurements 1 week post operation. **B**) Simultaneous longitudinal *in vivo* assessment of ^99m^Tc-MDP accumulation and bone volume in the created bone defects. Scale bar = 4 mm. Data is depicted as mean ± SEM (n = 5).

Even though *in vivo* micro-CT is a powerful mean to assess kinetics and spatiality of bone formation, it only captures the very last step of bone formation, namely the presence of densely mineralized areas. Since the formation of these areas relies on the continuous deposition of hydroxyapatite (Ca_10_(PO_4_)_6_(OH)_2_), also called bone mineral, we hypothesized that the accumulation of orthophosphates (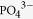) could be used to determine areas of bone formation at earlier time points. In order to test this, ^99m^Tc-MDP was injected into operated mice and its distribution was non-invasively monitored by SPECT. Bisphosphonate tracers were shown to adsorb to hydroxyapatite and have been previously applied to visualize bone and joint disorders using SPECT^21^. At 1 week of treatment, injection of ^99m^Tc-MDP led to its subsequent accumulation in many metabolically active anatomical areas, such as joints, teeth, vertebrae, and also the created bone defects (Fig. 3A). In the samples that were treated with BMP-2-containing hydrogels, longitudinal *in vivo* evaluation by SPECT shows ^99m^Tc-MDP signals in the bone defects already after 1 week of treatment (Fig. 3B). Interestingly, very high levels of activity are obtained at 2 weeks of treatment, a time point where only a limited amount of bone was previously detected by micro-CT indicating that monitoring of phosphate accumulation might serve as predictor of bone growth. Although the ^99m^Tc-MDP signals as well as the increase in bone volume reached a maximum after 4 weeks of treatment, significantly high SPECT activities remained up to week 12, clearly demonstrating the timely different behavior of micro-CT analysis and SPECT evaluations.

### Temporal and spatial *in vivo* assessment of hydroxyapatite deposition during bone defect regeneration – anterior-posterior axis

**Figure 4:**
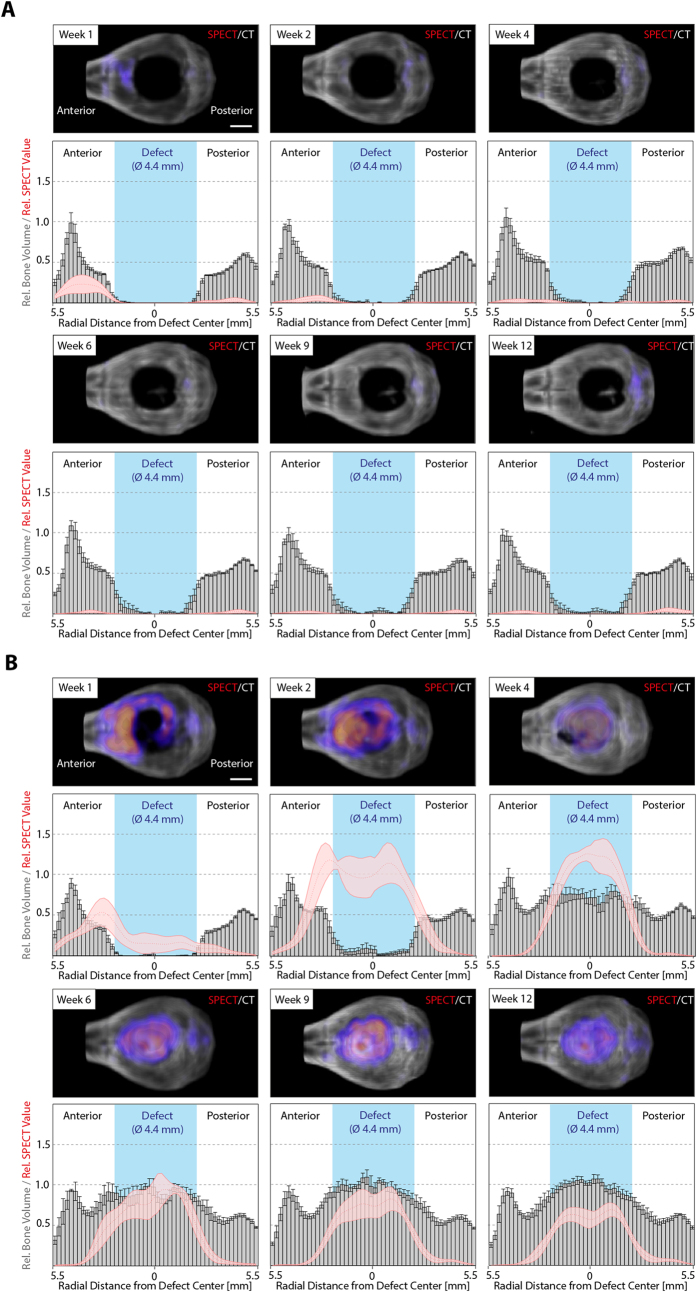
Course of mineralization and ^99m^Tc-MDP accumulation during bone defect healing from the dorsal view. *In vivo* SPECT micro-CT measurements were performed to localize ^99m^Tc-MDP accumulation at indicated time points after operation for (**A**) empty control gels and (**B**) BMP-2 loaded hydrogels. Upper panels display representative images of merged 3D volume rendered micro-CT (grayscale) and SPECT (pseudo colored) measurements (upper panels). Lower panels show histograms depicting the radial distribution from the defect center of relative bone volume (gray bars) and SPECT values (red line). Data analysis and presentation were performed as described in the methods section. Scale bars = 2 mm. Data is depicted as mean ± SEM (n = 5).

Since ^99m^Tc-MDP signal was accumulating in healing bone defects, which under BMP-2 treatment are areas of active bone mineral deposition, we next aimed at longitudinally localizing zones of active bone formation and remodeling, as well as correlating these zones to zones of bone mineral deposition. For bone defects treated with empty control hydrogels, after one week post-implantation a minimal SPECT signal was observed, which resulted in a small thickening of the bone at the edge of the defect as can be seen at week 2 (Fig. 4A). Thereafter no significant SPECT signal as well as no increase in bone volume was observed. In contrast, the BMP-2 hydrogel samples induced a comparably stronger signal on the anterior side of the defect and already an observable signal inside the bone defect (Fig. 4B). At 2 weeks the SPECT signal strongly increased in the surrounding of the defect as well as inside the defect. Interestingly, at this stage, the highest signals were still observed at the implant-bone interface, while later (weeks 4 to 12) the maximum intensity had shifted towards the center of the defect. Additionally, the longitudinal co-evaluation of bone healing using micro-CT and SPECT indicates that in the initial healing phase (weeks 1 to 4) the SPECT signal precedes deposition of bone. Later in bone regeneration (weeks 6 to 12) the absolute bone volume in the defect area remains constant, suggesting that no additional bone mineral is being deposited. However, the strong SPECT signal detected in the center of the defect indicated that novel hydroxyapatite was still being formed. This can well be explained by ongoing bone remodeling of the initial bone structures, which describes the last phase of bone regeneration.

### Temporal and spatial *in vivo* assessment of hydroxyapatite deposition during bone defect regeneration – dorsal-ventral axis

**Figure 5:**
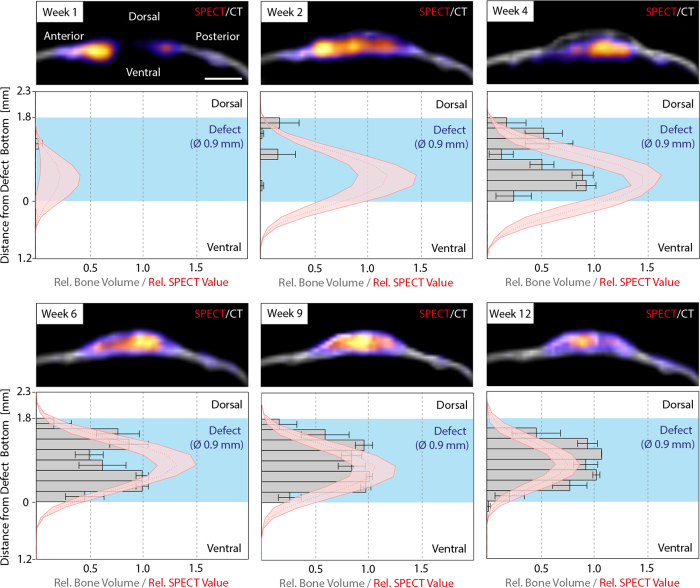
Course of mineralization and ^99m^Tc-MDP accumulation during bone defect healing from the sagittal view. ^99m^Tc-MDP and bone volume distribution on the dorsal-ventral axis were monitored by combined *in vivo* SPECT and micro-CT measurements within a cylindrical region (Ø 0.9 mm) in the center of the defect. Upper panels display representative images of merged 3D volume rendered micro-CT (grayscale) and SPECT (pseudo colored) measurements (upper panels) from a sagittal view. Lower panels show histograms depicting the dorsal-ventral distribution of bone volume (grey bars) and SPECT values (red line). Scale bar = 2 mm. Data is depicted as mean ± SEM (n = 5).

To assess the course of hydroxyapatite deposition in the hydrogel implant relative to the dorsal-ventral axis, total ^99m^Tc-MDP signals and bone volume were quantified within a cylinder of a diameter of 0.9 mm in the middle of the defect. This showed that the previously seen mineral deposition appearing at week 2 predominantly occurred on the dorsal side of the implant, leading to a thin upper bone plate on top of the hydrogel (Fig. 5). Not before 4 weeks post-operation, mineralization on the ventral side was detected and only at week 6 the hydrogels became homogeneously mineralized. Interestingly, at week 1, 2, and 4 the SPECT signal was strongest on the ventral side of the implant whereas only marginal activity was detected on the dorsal side suggesting that after the formation of the initial bone plate no significant hydroxyapatite deposition occurred on the dorsal side. From week 6 on, the peak of the SPECT signal transitioned to the center of the defect, coinciding with the previous hypothesis that from week 6 on the deposited hydroxyapatite was associated with bone remodeling rather than *de novo* bone formation.

### *Ex vivo* spatial determination of cellular ingrowth into the hydrogel implant and hydrogel implant remodeling

**Figure 6:**
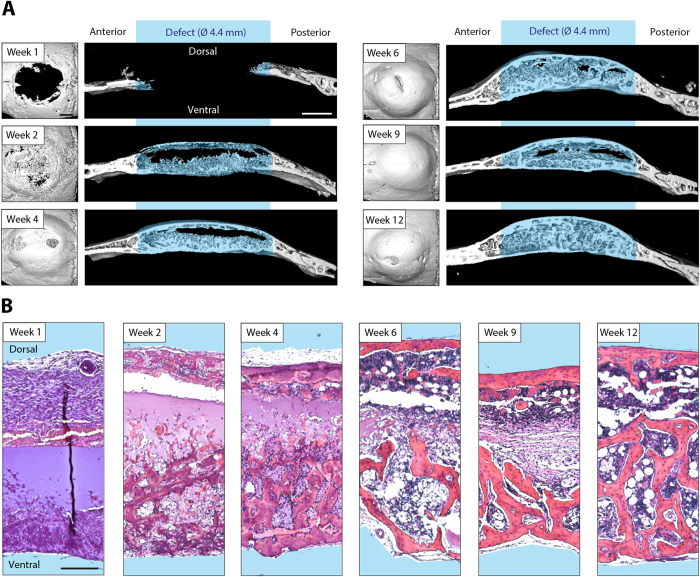
*Ex vivo* analysis of cell invasion, mineralization and bone remodeling in BMP-2 presenting calvarial hydrogel implants. Tissue specimens were harvested at indicated time points for *ex vivo* analysis. **A**) 3D surface rendered micro-CT measurements (dorsal view and sagittal view, scale bars = 1 mm) and **B**) hematoxylin and eosin stained sections are shown for each time point (scale bar = 200 μm).

The longitudinal evaluation of ^99m^Tc-MDP accumulation suggests that radioactively labeled phosphate can be applied to localize *in vivo* sites of active bone formation including *de novo* formation as well as remodeling of existing structures. In order to confirm this, samples of treated bone defects were harvested at time points post operation corresponding to the ones of the longitudinal analysis. The previous longitudinal evaluation of bone mineral deposition by SPECT and micro-CT indicated that the formation of bone tissue in the calvarial defects originated from a lower and an upper healing front, both of which forming a bony layer over the implant material. Indeed, assessment of end point micro-CT images show that two weeks post operation a bone layer can be detected on the dorsal and the ventral side of the implant (Fig. 6A,B). However, whereas the dorsal layer consisted of a mostly continuous bone plate, the bottom part was composed of small trabecular bone structures that commenced to infiltrate the hydrogel implant. This coincided with the previously observed strong SPECT signal on the ventral side of the implant indicating that at week 2 post operation hydroxyapatite deposition primarily occurred on the ventral side. A comparable pattern, which is as well in accord with the longitudinal SPECT and micro-CT measurements, was observed at week 4. The ventral trabecular structures had further advanced into the hydrogel implant and the dorsal bone plate had thickened. Moreover, we showed in the longitudinal assessments that the increase in bone volume seemed not to be limited to the defect, but that as well the wound edges significantly increased in volume from week 1 to week 4. Indeed, the *ex vivo* end point micro-CT measurements at week 2 and week 4 confirmed this, showing thick bone structures at the wound edge compared to the original calvarial bone as measured at week 1. At week 6, the hydrogel implant was evenly covered by bone structures and the original implant material was almost completely replaced by ingrown tissue. Of note, the spaces between trabecular bone structures started to exert characteristic features of bone marrow-like tissue. From week 6 to 12, the ventral and dorsal bone surfaces didn’t exhibit considerable changes and only trabecular structures in the defect were remodeled. Moreover, whereas at week 6 and 9 still parts of the hydrogel implant existed that were covered by connective tissue-like structures or initial hydrogel material, these areas were resorbed by week 12 and the defect was fully remodeled. As suggested by the longitudinal measurements, the hydrogel implant had been infiltrated from the ventral side and after 12 weeks it had been completely replaced with a bone shell on the outside of the implant and trabecular interwoven bone therein.

## Discussion

This paper describes the longitudinal characterization of biomaterials-induced bone formation in small laboratory animals by *in vivo* multiplexed multi-pinhole SPECT and micro-CT co-registration and evaluation. This approach allows the longitudinal characterization of active bone formation by the determination of localized metabolic imaging. In comparison to evaluations based on micro-CT alone, the combination of *in vivo* micro-CT and metabolic imaging has several advantages: i) the bone forming activity can be seen very early in the process, ii) it can be applied for biomaterials (e.g. hydroxyapatite/tricalciumphosphate) with a radiodensity similar to that of native bone and iii) sites of bone remodeling can be followed. This makes hybrid micro-CT and nuclear imaging a powerful combination for assessing various bone healing implants and selecting ideal time points for in depth *ex vivo* analysis (e.g. histologies or functional assays) without the need for longitudinal sampling of tissues.

Here, we apply a recently developed PEG-based material, which does not promote bone regeneration in absence of an osteogenic signal, but when supplemented with BMP-2 has been shown to guide bone formation in rat critical sized defects after two weeks of treatment^16^. As this material contains MMP-1 degradable linkers in its backbone, it is designed to be provisional and release the entrapped BMP during host tissue ingrowth. Of note, the degradation kinetics of the material does not only affect the release of the entrapped growth factor, but was also shown to have a significant influence on the sequence and progression of bone formation and remodeling^16^. Soft hydrogels (1.7%) as used in this study are easily amenable for infiltration by cells of the early healing cascade and are continuously replaced by endogenous bone tissue, leading to full coverage of the critical-sized defects created in C57BL/6 mice by 12 weeks of treatment (Figs. 1 and 2). Thus, in addition to providing an ideal system to induce bone defect regeneration, they allow to gather spatiotemporal information on the bone formation cascade *in vivo*.

Sites of active bone remodeling can be visualized and quantified by bone specific uptake of ^18^F-fluoride (^18^F) or hydroxyapatite binding bisphosphonates with chelated ^99m^Tc and the imaging by PET and SPECT, respectively^14^. Both approaches are commonly used for the evaluation of bone related disorders such as inflammation and bone metastasis in clinical settings^22^. While the two procedures are comparable regarding the determination of areas of bone turnover and metabolic activity, SPECT due to cost issues and ease of tracer production has been used more frequently^10^. Recently both technologies have been employed to evaluate the healing of bone by tissue engineering approaches. For example SPECT was successfully used to monitor the regeneration of bone tissue after transplantation of pre-differentiated and pre-vascularized mesenchymal stem cell-based tissue constructs^23^. However, while in another study the healing of bone defects after application of BMP releasing biomaterials could be followed by PET, SPECT has not provided data of sufficient quality^13^.

In our current study, hybrid multi-pinhole SPECT/micro-CT imaging has been employed, allowing the longitudinal scanning of the healing defects with good spatial resolution and good contrast-to-noise ratio^15^. The used pinhole technology compared to the clinical applied parallel hole collimators allows to achieve a higher resolution at the expense of sensitivity. To improve the sensitivity we used 4 detectors each with a nine pinhole collimator resulting in a sensitivity of about 1900 cps/MBq and a resolution of about 0.6 mm. This allows following the location of the metabolic rate of new bone tissue formation with a remarkable quality. Due to the much higher sensitivity of Nuclear Imaging technologies compared to CT or MRI the biology can be visualized much earlier and enables to visualize the biological events in living animal in a very early stage in a longitudinal manner. This helps to gain a better understanding of the biology *in vivo* and to reduce the number of animals.

Our data indicate that one week after treatment, the injected ^99m^Tc-MDP accumulates in bone areas surrounding the defects (Fig. 4A,B) correlating well with the trauma-initiated bone remodeling in the pre-existing bone seen in other studies assessing bone regeneration in calvarial defects^24^. While bone defects treated with control hydrogels do not show any metabolic labeling, defects treated with BMP releasing hydrogels accumulate substantial quantities of the ^99m^Tc-MDP after one week, even at sites that do not yet contain sufficient quantities of bone mineral to be detected by micro-CT. This correlates with the notion that ^99m^Tc-bisphosphonates associate with osteoblastic activity^25^,^26^,^27^. Dorsal view images show that zones of active bone formation start, as expected, at the defect edge and from two weeks after treatment on are distributed throughout the whole defect (Fig. 4B). Interestingly, on the sagittal view, after the very early onset of metabolic activity at the anterior edge of the wound, the activity is distributed throughout the whole ventral part of the defect site. This correlates with micro-CT and histological observations where a ventral and dorsal bone layer is detected after two weeks of implantation. From two weeks of implantation on, we observed the ingrowth of cells and then bony structures from the whole ventral plate into the hydrogel, which by proteolytic activity is fully resorbed by 12 weeks of implantation. Another important observation is the continuous uptake of the metabolic tracer inside the healing defect after reaching a constant amount of bone mineral by *in vivo* micro-CT evaluation. This clearly indicates ongoing bone remodeling, which is reflected in active bone formation that does not result in an increase in bone mass. Micro-CT and histological evaluation demonstrate that small bony structures observed in the early deposited bone (4 weeks) merge to larger trabecular structures after 12 weeks of treatment.

In order to account for different effective tracer concentrations in individual measurements, SPECT signals were standardized by a semi-automated MATLAB script, using areas in the dental pulps as internal invariable reference. For the evaluation of SPECT and CT signals from dorsal view, concentric anterior and posterior areas (relative to the center of the defect) were selected to account for distances from the defect edge and differences due to anatomical location within the skull. Although this approach is a first step towards the unbiased standardization of measurements of different animals and time points, more rigorous standardization, such as tightly controlling the dose of the administered tracer and the reproducible positioning of the sites of interest are expected to improve the readout parameters. Additionally, the development of novel artifact-free overlapping multi-pinhole systems as well as collimators with higher resolution and sensitivity would further enhance spatial resolution and signal-to-noise ratio while preserving sensitivity^28^. As this study was designed to show the potential of currently available SPECT technology to longitudinally follow active bone formation, it has been restricted to one animal model and one material composition that contained or did not contain entrapped BMP-2. However, we strongly believe that the broad applicability of this method makes it well suitable for further investigations to study and tailor the properties of biomaterials as well as for different bone healing models.

## Conclusion

The hybrid evaluation of longitudinal *in vivo* micro-CT and ^99m^Tc-MDP based SPECT is shown to provide spatial information on bone forming and remodeling. It allows the reduction of animal numbers by improving the value of acquired data through the use of animals as their own control as well as the determination of optimal time points of tissue sampling for the acquisition of high resolution images, immunohistochemical or functional analysis. Together with the implementation of tracers that allow the concomitant evaluation of materials stability and the release of growth factors, combined ^99m^Tc-MDP based SPECT and micro-CT evaluations hold great promise for the study and optimization of bone inducing biomaterials.

## Methods

### Formation of disc-shaped hydrogel implants

Metalloprotease (MMP)-sensitive TG-PEG hydrogels were prepared as described previously (Ehrbar Biomacromolecules). In brief, eight-arm PEG precursors containing the pending factor XIIIa substrate peptides glutamine acceptor (n-PEG-Gln) or lysine donor with an additional MMP-sensitive linker (n-PEG-*MMP*_*sensitive*_-Lys) were mixed stoichiometrically (final dry mass content 1.7%) in Tris-Buffer (50 mM, pH 7.6) containing 50 mM calcium chloride. 50 μM Lys-RGD peptide and if indicated 2 μg BMP-2 (produced as described previously^29^) were added to the precursor solution prior to initiation of cross-linking by 10 U/mL thrombin-activated factor XIIIa and vigorous mixing. 24 μl of this mixture were then sandwiched between sterile hydrophobic glass microscopy slides (obtained by treatment with SigmaCote) separated by spacers (ca. 1.25 mm thickness) and clamped with binder clips. After incubating the forming matrices at 37 °C for 30 min, hydrogel discs were released from glass slides and stored in humidified atmosphere until application.

### Calvarial defect model

*In vivo* experiments were approved by the veterinary offices of the cantons Zurich and Aargau and were conducted in accordance with the Swiss law of animal protection. Adult wild-type C57BL/6 female mice (purchased from Harlan) at the age of 10-12 weeks at the start of the experiments were used. A craniotomy with a diameter of 4.4 mm was created using a core drill. Preformed TG-PEG hydrogel discs with a diameter of 4.4 mm and a height of 1.25 mm were placed in the defect and the skin was closed with sutures. Animals were sacrificed at indicated time points post-operation for *ex vivo* end point analysis by micro-CT and histology.

### Ex vivo micro-CT and histological analysis

After fixation in formalin and storage in PBS, complete skulls were scanned in a micro-CT 40 (Scanco Medical AG) operated at energy of 70 kVp and intensity of 114 μA. Scans were executed at a high-resolution mode resulting in a voxel size of 10 μm. In reconstructed images bone tissue was segmented from background using a global threshold of 12% of maximum gray value. A cylindrical mask with a diameter of 4.4 mm was manually placed at the bone defect. Bone volume and connectivity were measured using the ImageJ plugin BoneJ^30^. Coverage was measured in a dorsoventral projection of the cylindrical mask. 3D surface rendered images were created using the ImageJ 3D viewer plugin^31^. For histological analysis, samples were decalcified using USEDECALC (MEDITE, cat. no. 40-3310-00) and embedded in paraffin. Sections (4 μm) were then stained with hematoxylin & eosin and images were acquired using a Zeiss 200M inverted microscope.

### Longitudinal *in vivo* micro-CT and SPECT measurements

The ^99m^Tc-MDP were prepared by adding 3 GBq of a 3 mL 99mTcO4- solution freshly eluted from a 99Mo/99mTc generator (Mallinckrodt Switzerland, Cham Switzerland) to a MPD-kit (Rotop, Dresden, Germany). The solution was diluted to the necessary activity concentration with 0.9% sodium chloride solution.

The mice were i.v. injected with 20-40 MBq in about 100 μL 0.9% sodium chloride solution. The mice were anesthetized with an initial dose of 4% Isoflurane and were reduced to 1.5 to 2% to maintain the anesthesia (breath rate was kept between 80 and 120 per minutes).

A NanoSPECT/CT plus (Bioscan, Washington, D.C., USA) was used for imaging. The CT was performed at tube voltage of 65 kV and tube current of 125 μA with duration of 20 min. The SPECT was performed with all four detectors installed with standard nine pinhole aperture (NSP-108-M14-WB; Bioscan, Washington, D.C., USA) with 30 angles with 60 s per angle. SPECT acquisitions were reconstructed via the manufacturers HiSPECT software using Ordered Subsets Expectation Maximization (OSEM) iterative reconstruction algorithm with 9 iterations and 4 subsets.

### Normalization, analysis and data presentation of longitudinal sets of *in vivo* micro-CT and SPECT measurements

In order to correct for changes in ^99m^Tc-MDP radioactivity and potential variations in the amount of injected ^99m^Tc-MDP, all grayscale stacks resulting from SPECT measurements were normalized to a cylindrical reference area in the anterior region of the mouse skull. This was done using an automated image analysis script written in MATLAB (R2013b, MathWorks Inc, USA) that assessed the accumulated amount of ^99m^Tc-MDP signal in this region, calculated the ratio to a predefined standard and linearly corrected all pixels of the recorded stack individually by this ratio. To correct for differences in positioning of the animals, all recorded stacks of micro-CT and SPECT measurements were rotated around x, y, and z axes until they were anatomically oriented in a predefined position using a semi-automated macro in ImageJ. Standardized volumes (length = 15 mm, width = 15 mm, height = 7.5 mm) comprising the defect were then extracted from the stacks. SPECT and micro-CT values in resulting normalized and oriented stacks were analyzed using a semi-automated script written in MATLAB. In brief, bone tissue in micro-CT images were segmented from background using a global threshold of 0.25 of maximum gray value. For the analysis of the anterior-posterior distribution, the defect was then manually localized and bone volume as well as SPECT signals were automatically assessed through the whole stack on concentric rings around the defect center. For data presentation, histograms were created showing the summed up micro-CT and SPECT values of each concentric circle for the anterior and posterior semicircles at each time point. For the analysis of the dorsal-ventral distribution, additionally the defect bottom was defined and bone volume and SPECT signals were assessed in a virtual dorsoventral cylinder with a diameter of 0.9 mm placed in the middle of the defect. Subsequently, recorded values from different animals were transformed along the z-axis to align the individual defect bottoms. For data presentation, histograms were created for each time point showing the summed up micro-CT and SPECT values of each plane of the z-stack. 3D volume rendered images were created using the ImageJ 3D viewer plugin^31^.

### Statistical Analysis

All mean values were compared by one-way analysis of variance (ANOVA) using MATLAB (R2013b, MathWorks Inc, USA) followed by a Tukey-Kramer *post hoc* test for pairwise comparison. Statistical significance was accepted for *p* < 0.05.

## Author Contributions

P.S.L, M.B. and M.E. designed the study, analyzed the data and wrote the manuscript; P.S.L, S.M., A.K. performed experiments; P.S.L., A.B., P.P., A.A. and B.R.P. performed data acquisition; P.C., F.E.W. and R.S. contributed reagents, analytic tools and scientific advice; All authors reviewed the manuscript.

## Additional Information

**How to cite this article**: Lienemann, P. S. *et al* Longitudinal *in vivo* evaluation of bone regeneration by combined measurement of multi-pinhole SPECT and micro-CT for tissue engineering. *Sci. Rep.* **5**, 10238; doi: 10.1038/srep10238 (2015).
