# Erratum: Longitudinal *in vivo* evaluation of bone regeneration by combined measurement of multi-pinhole SPECT and micro-CT for tissue engineering

**DOI:** 10.1038/srep12391

**Published:** 2015-11-12

**Authors:** Philipp S. Lienemann, Stéphanie Metzger, Anna-Sofia Kiveliö, Alain Blanc, Panagiota Papageorgiou, Alberto Astolfo, Bernd R. Pinzer, Paolo Cinelli, Franz E. Weber, Roger Schibli, Martin Béhé, Martin Ehrbar

Scientific Reports 5: Article number: 1023810.1038/srep10238; published online: 05 19 2015; updated: 10 20 2015

This Article contains errors.

Affiliation 6 was incorrectly listed as ‘Department of Chemistry and Applied Biosciences, ETH Zurich, 8092 Zurich, Switzerland’. The correct affiliation is listed below:

Department of Chemistry and Applied Biosciences, ETH Zurich, 8093 Zurich, Switzerland.

In the Results section under subheading ‘Longitudinal in vivo assessment of biomaterial-induced bone formation’

“Over the following 8 weeks the plural stayed closed”.

should read:

“Over the following 8 weeks the defects stayed closed”

Under subheading ‘Temporal and spatial in vivo assessment of hydroxyapatite deposition during bone defect regeneration – anterior-posterior axis.’

“Thereafter no significant SPECT signal as well as no increase in bone volume was observed. In contrast, the BMP-2 hydrogel samples induced a comparably stronger signal on the anterior side of the defect and already an observable signal inside the bone defect (Fig. 4B).”

should read:

“No significant SPECT signal as well as no increase in bone volume were observed thereafter. In contrast, after 1 week of treatment BMP-2 hydrogel samples induced a comparably stronger signal on the anterior side of the defect and already an observable signal inside the bone defect (Fig. 4B).”

In the Discussion section

“The used pinhole technology compared to the clinical applied parallel hole collimators allows to achieve a higher resolution at the expense of sensitivity.”

“should read:”

“In comparison to the clinically applied parallel hole collimators the herein used pinhole technology allows to achieve a higher resolution at the expense of sensitivity.”

In addition, the Acknowledgements section,

“This work has been supported by funding from Swiss National Science Foundation (Nos. 31003A_141051, CR2313_143766, CR3213_125426), and the Center for Clinical Research, University Hospital Zurich and University of Zurich.”

should read:

“This work has been supported by funding from Swiss National Science Foundation (Nos. 31003A_141051, CR2313_143766, CR3213_125426), the European Unionn’s Seventh Framework Programme (iTERM grant agreement No. 607868) and the Center for Clinical Research, University Hospital Zurich and University of Zurich.”

In the References section

Iagaru, A., Mittra, E., Dick, D. W. & Gambhir, S. S. *Prospective evaluation of (99m)Tc MDP scintigraphy, (18)F NaF PET/CT, and (18)F FDG PET/CT for detection of skeletal metastases. Mol. Imaging Biol.* **14**, 252–259 (2012).

should read:

Iagaru, A., Mittra, E., Dick, D. W. & Gambhir, S. S. *Prospective evaluation of (^99^m)Tc MDP scintigraphy, (^18^)F NaF PET/CT, and (^18^)F FDG PET/CT for detection of skeletal metastases. Mol. Imaging Biol.* **14**, 252–259 (2012).

And lastly,

Park-Holohan, S. J., Blake, G. M. & Fogelman, I. *Quantitative studies of bone using (18)F-fluoride and (99m)Tc-methylene diphosphonate: evaluation of renal and whole-blood kinetics. Nucl. Med. Commun.* **22**, 1037–1044 (2001).

should read:

Park-Holohan, S. J., Blake, G. M. & Fogelman, I. *Quantitative studies of bone using (^18^)F-fluoride and (^99^m)Tc-methylene diphosphonate: evaluation of renal and whole-blood kinetics. Nucl. Med. Commun*. **22**, 1037–1044 (2001).

